# Anti-inflammatory and anti-angiogenic effects of *Withania somnifera* extract on liver toxicity induced by silver nanoparticles in vivo

**DOI:** 10.25122/jml-2024-0050

**Published:** 2024-07

**Authors:** Abdullah Asser Ahmed Alghamdi, Eman Abdallah Ahmed Abdallah, Mohamed Farouk El-Refaei

**Affiliations:** 1 Department of Biology, Faculty of Science, Al-Baha University, Al-Baha, Saudi Arabia; 2 Faculty of Medicine, Al-Baha University, Al-Baha, KSA; 3 Department of Forensic Medicine and Clinical Toxicology, Faculty of Medicine, Zagazig University, Zagazig, Egypt; 4 Department of Biochemistry and Molecular Biology, Genetic Institute, Sadat City University, Sadat City, Egypt

**Keywords:** *W. somnifera*, silver nanoparticles, antioxidants, NF-κB, VEGF, cytokines

## Abstract

The liver is a critical organ in the human body and is frequently exposed to numerous exogenous toxic substances, including silver nanoparticles (AgNPs). This study aimed to examine the anti-inflammatory, anti-angiogenic, and hepatoprotective effects of *Withania somnifera* (*W. somnifera*) extract on AgNP-induced liver toxicity in Swiss mice. Fifty mice were divided into five groups. Group I (negative control) consisted of ten mice. Group II received oral *W. somnifera* extracts (80 mg/kg/bw) for 14 days. Group III was injected intraperitoneally (i.p.) with AgNPs at a daily dose of 35 mg/kg/bw for 3 days. Group IV received i.p. AgNPs for 3 days, followed by saline for 14 days. Group V received i.p. AgNPs for 3 days, followed by oral *W. somnifera* (80 mg/kg/bw) for 14 days. Liver function tests, pro and anti-inflammatory cytokines, antioxidant activities, protein carbonyl (PC) levels, liver histopathological analysis, immunohistochemical expressions of transcription factor (NF-κB), and vascular endothelial growth factor (VEGF) were examined. Group III had elevated levels of liver function, a significant increase of pro and anti-inflammatory cytokines, antioxidant activity, and PC levels. Histological observations revealed congested sinusoids filled with red blood cells (RBCs) and hepatocyte necrosis. Also, positive expressions of NF-κB and VEGF were detected compared with Group I. However, the administration of *W. somnifera* to Group V revealed significant changes with evident improvements in liver function biomarkers, pro and anti-inflammatory cytokines, antioxidant activities, oxidative stress markers (PC), and histopathological and immunohistochemical parameters compared to Group III. The results revealed that *W. somnifera* has promising and potential hepatoprotective, anti-inflammatory, and anti-angiogenic effects against liver toxicity. Further detailed studies are recommended to explore the potential of *W. somnifera* as a treatment for human liver ailments.

## INTRODUCTION

The liver is the primary organ responsible for metabolism and detoxification, processing toxins and xenobiotics. It has antioxidant capabilities and plays a crucial role in glycogen storage and protein synthesis [1]. Additionally, the liver acts as a biological barrier by isolating and removing various foreign substances. However, it is a potential target organ for nanomaterials (NMs), associated with oxidative stress, inflammatory stress, liver dysfunction, DNA damage, steatosis, fibrosis, and cell death [2].

Nanoparticles (NPs), which have dimensions ranging from 1 to 100 nanometers [3], primarily enter cells through endocytosis, leading to the formation of endocytic vesicles and the release of ions into the cytoplasm [4]. In particular, silver nanoparticles (AgNPs) can penetrate cell membranes, alter cell structures, and potentially induce cell death. In addition, the release of silver ions has the potential to alter DNA replication, produce reactive oxygen species, and increase cell membrane permeability [5].

Silver nanoparticles are found in various consumer products, including laundry machines, dusting cloths, food, and personal hygiene products [6]. As a result, NPs found in daily home objects are frequently dumped straight into sewage systems, potentially contaminating rivers. AgNPs are known for their antibacterial qualities, which allow them to be used in coatings for fabric and other textiles, food storage equipment, and cosmetics [7]. They are also found in disinfectants, wound dressings, central venous catheters, and surgical nettings, as well as in other public health use and medical items. According to various studies, AgNPs may also have cytotoxic characteristics because they cause a typical cellular reaction of reactive oxygen species (ROS) production [8]. The concentration of silver in human blood is less than 2.3 µg/L [9]. Silver binds to red blood cells and globulins and is mostly stored in the liver, brain, and reticuloendothelial system. Silver is predominantly stored in the liver, brain, and reticuloendothelial system, with most being eliminated through feces and a small amount through urine and sweat (8%) [10]. The liver, which retains up to 80% of NPs in the blood, acts as a biological filtration system. However, NP accumulation can impact normal liver functions by altering hepatic cell structure and function [11].

Cytokines play a significant role in liver inflammatory diseases by regulating tissue homeostasis and promoting immune responses to injury and disease [12]. These cytokines drive many inflammatory liver conditions, often leading to fibrosis and cirrhosis [13]. Vascular endothelial growth factor (VEGF) is a key factor in angiogenesis, closely associated with inflammation and hypoxia. Liver disorders that cause inflammation and hypoxia lead to elevated levels of VEGF. Persistently high VEGF levels increase the risk of chronic liver disorders, such as hepatic viral infections, fatty liver diseases, liver cirrhosis, and, ultimately, hepatocellular cancer. If inflammation and hypoxia persist, they can continuously stimulate VEGF production, exacerbating these liver conditions and increasing the potential for severe complications [14].

Natural plant products have long been valued for their usefulness in treating human illnesses, with interest in studying and isolating active ingredients in plants dating back to prehistoric times [15]. *Withania somnifera* (*W. somnifera*), a member of the Solanaceae family, has long been used as a medicinal plant. It is naturally found in many parts of the world, mainly in drier areas, subtropical zones of the Mediterranean, northern Africa, and south-west Asia. Over the years, *W. somnifera* has gained recognition for its safe and effective use in promoting mental and general health without any negative side effects [16,17]. *W. somnifera* extract also contains various chemical constituents, including terpenoids, alkaloids, flavonoids, tannins, and saponins [18]. The active ingredients responsible for its therapeutic benefits include withanolides (withaferin A and D) as well as tripolin, anaferine, anhygrine, and withanine. Numerous studies have characterized the biological and pharmacological characteristics of *W. somnifera*, demonstrating its antimicrobial, anti-inflammatory, antioxidant, antiulcer, and antidepressant properties [19, 20]. The present study aimed to evaluate the anti-inflammatory, anti-angiogenic, and hepatoprotective properties of *W. somnifera* extract. We hypothesized that the extract from *W. somnifera* could offer potential benefits in mitigating the hepatic toxicity caused by AgNPs.

## MATERIAL AND METHODS

### Silver nanoparticles (AgNPs)

Silver nanopowder with a particle size of less than 100 nm (7440-22-4) and a 99.9% trace metal basis was obtained from Sigma-Aldrich. The AgNPs were coated with carbon after being dissolved in 0.5% aqueous carboxymethylcellulose (Sigma-Aldrich). However, for systemic circulation, AgNPs were sonicated for 10 minutes before injection to ensure even dispersion. The size of the nanoparticles was checked using an electron microscope [21].

### Experimental animals

Fifty adult male Swiss mice weighing 23–25 g were included in this study, obtained from the animal house of King Abdulaziz University in Jeddah, Saudi Arabia. The mice were allowed to acclimate to the study site (Faculty of Medicine, Al-Baha University) for five days under ideal environmental conditions: 12-hour light-dark cycles, room temperature (20 ± 2°C), moderate humidity (60 ± 5%), with unlimited access to food and water.

### Experimental design and administration of AgNPs

**Figure 1:**
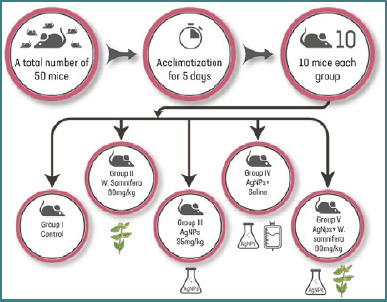
Schematic diagram for experimental design and doses

Fifty mice were randomly assigned to five groups. Group I (negative control) consisted of ten mice (Figure 1). Group II received oral *W. somnifera* extract (80 mg/kg/ body weight) for 14 days. Group III mice were injected intraperitoneally (i.p.) with AgNPs dissolved in distilled water at a daily dose of 35 mg/kg/bw for 3 days [22]. Group IV received i.p. AgNPs were administered for 3 days, followed by saline for 14 days, while Group V received i.p. AgNPs for 3 days, followed by oral *W. somnifera* (80 mg/kg/bw) for 14 days. Twenty-four hours after the last treatment, the mice were euthanized through cervical dislocation. Blood was collected from the heart for serum analysis of liver function parameters and cytokines: interleukin-1 beta (IL-1β), tumor necrosis factor-alpha (TNF-α), interleukin-6 (IL-6), and interleukin-10 (IL-10). The liver was cleaned and dissected into two parts: one part was used to determine the activity levels of superoxide dismutase (SOD), glutathione peroxidase (GSH-Px), catalase (CAT), and malondialdehyde (MDA), a product of lipid peroxidation and oxidative stress aspect (PC) in liver homogenate. The other part was kept in 10% formalin for immunohistochemical and histopathological examinations.

### Withania somnifera (W. somnifera) extract

*W. somnifera* root parts were collected from the Al-Baha region of Saudi Arabia from June to August 2022. The samples were classified taxonomically at the Botany Department, Faculty of Science, Al-Baha University. The plant materials were dried in the dark at room temperature.

About 1 g of *W. somnifera* root powder was added to 100 ml of triple-distilled water in a 500 ml Erlenmeyer flask, allowed to sit overnight, and then heated to reduce the volume to about one-fourth. The mixture was cooled to ambient temperature and filtered through Whatman filter paper to produce a light brown aqueous extract. This extract was further concentrated under a vacuum to obtain a dry form redissolved in water to a final concentration of 80 mg/kg body weight [23].

### Toxicity study in Swiss Albino mice

Male Swiss albino mice were treated orally with different concentrations (200, 600, 1000, and 1400 mg/kg/bw) of *W. somnifera* extract. The LD50 was estimated to be 1050 mg/kg body weight. The test was applied to determine LD50 using the percentages of deaths per group versus doses’ log values [24].

### Quantitative determination of IL-1β, TNF-α, IL-6 and IL-10 cytokines

Cytokines regulate immune responses to inflammation, trauma, and infection. Certain cytokines act as pro-inflammatory agents, aggravating disease, while others act as anti-inflammatory agents, reducing inflammation and accelerating healing. The anti-inflammatory cytokines IL-6 and IL-10, as well as the pro-inflammatory cytokines IL-1β and TNF-α, were measured in mouse serum using commercially available ELISA kits, strictly adhering to the manufacturer’s instructions. Mouse serums were diluted (1:2) for quantitative cytokine testing: IL-1β (ab22944), TNF-α (ab208348), IL-6 (ab222503), and IL-10 (ab100697). Monoclonal antibodies against the corresponding cytokine were used to apply internal controls and test standards on a solid phase. Following incubation and washing, a peroxidase conjugate (second anti-species antibody) was added to create a cytokine complex. To get rid of the unbound conjugate, a second wash was applied. The presence of cytokines was indicated by a color reaction after adding a chromogenic substrate to the enzyme. Absorption was measured at 450, 570, and 620 nm using a BIO-RAD reader and was proportional to the cytokine concentration. The sample data was then analyzed using the standard curve for each cytokine [25].

### Determination of serum liver function

The blood samples were centrifuged at 3000 rpm for 15 minutes and then divided into smaller amounts for various analyses. Blood aspartate transaminase (AST), alanine transaminase (ALT), and alkaline phosphatase (ALP) levels were estimated using MAK052-1KT, MAK055-1KT, and SCR-004 colorimetric assay kits, respectively. The Sigma-Aldrich kits were utilized according to the manufacturer’s protocols [26]. Total protein was determined by the Biuret method [27], albumin by the bromocresol green method [28], and bilirubin was estimated by the method described by Jendrassik and Grof. Total bilirubin determinations were performed using Diamond Diagnostics kits [29,30]. To measure serum gamma-glutamyltransferase (GGT) activity, a kinetic method based on the measurement of transpeptidase activity was employed, as developed by Szasz [31].

### Determination of SOD, GSH-Px, CAT, MDA, and PC activity in liver homogenate

Each mouse’s liver was dissected, cleaned in phosphate-buffered saline (PBS), and homogenized on ice in a buffer containing 0.05 M Tris-HCl pH 7.9, 25% glycerol, 0.1 mM EDTA, and 0.32 M (NH4)2SO4, supplemented with a protease inhibitor tablet (Roche). Homogenization was performed using a Polytron homogenizer, followed by sonication in an ice bath for 15 seconds to prevent overheating. Next, it was centrifuged at 12000 rpm at 4°C for 5 minutes. A BCA kit (Pierce, Rockford, USA) was used to measure the protein concentration after the supernatant was divided into smaller portions and kept at -80°C. A lysis buffer diluted albumin was used as the standard. SOD and GSH-Px levels were measured using established protocols [32]. CAT activity was determined using the Claiborne method. The reaction mixture contained 50 µl of the sample, 19 mM H2O2, and 50 mM potassium phosphate buffer (pH 7.0). H2O2 was added to start the reaction, and absorbance changes were monitored for 30 seconds at 240 nm (2°C–5°C). CAT activity was expressed as units per gram of liver tissue, and one unit was defined as one µmol of hydrogen peroxide consumed per minute [33]. One of the main byproducts of lipid peroxidation is malondialdehyde. It reacts with thiobarbituric acid and other byproducts to produce a colored product that absorbs maximally at 535 nm, the color produced by all the substances that are reactive with thiobarbituric acid [34]. Protein carbonyl assay (PC) levels were assessed based on the measurement of carbonyl groups using 2,4 dinitrophenylhydrazine (DNPH). 800 µl of DNPH was combined with two hundred microliters of each protein extract. To precipitate the proteins, we added 1 ml of trichloroacetic acid (TCA 20%) and incubated the tubes for 1 hour at room temperature in the dark. The tubes were placed on ice for 10 minutes and centrifuged for 5 minutes at 4000. The optical density was measured at 340 nm [35].

### Histopathological examination of the liver

The liver specimens were dehydrated, embedded in paraffin, fixed in 10% paraformaldehyde, and sectioned at a thickness of 5 µm. The sections were stained with hematoxylin and eosin (H&E). Morphological changes were examined using an Eclipse 80i microscope (Nikon), and images were captured with a DS-Fi1 digital microscope camera (Nikon) [36].

### Immunohistochemical examination of NF-κB

The expression levels of NF-κB p65 in liver sections from all mouse groups were assessed using immunohistochemistry. The prepared liver sections were completely covered for one and a half hours with optimally diluted mouse monoclonal anti-NF-κB p65 [E379] (ab32536) at 1:50 dilutions in phosphate-buffered saline. The sections were then washed and exposed for 30 minutes to a secondary antibody from Sigma-Aldrich labeled with poly horseradish peroxidase enzyme. The 3,3′-diaminobenzidine tetrahydrochloride solution was then applied to the slides for 5 minutes. A nonaqueous permanent mounting medium called dibutyl phthalate xylene was used to mount the slides after they were cleaned and treated with Mayer’s hematoxylin. The slides were then dehydrated in graded alcohol (50%, 70%, 90%, and 100%), cleared with xylene, and washed again [37].

### Immunohistochemical examination of VEGF

Immunohistochemistry was employed to evaluate VEGF expression in the liver tissue sections. Formalin-fixed and paraffin-embedded tissue blocks were sectioned at 4 µm thickness and deparaffinized. Sections were rehydrated in ethanol for immunohistochemical staining. The sections were treated with 0.3% hydrogen peroxide in methanol for 30 min, normal horse serum for 30 min at room temperature, and then anti-VEGF polyclonal antibody (ab46154) for overnight incubation at 4°C (Sigma-Aldrich) diluted at 1:100. The avidin with biotin–labeled peroxidase complex technique was used to identify bound antibodies using a commercial kit, as suggested by the manufacturer (Sigma-Aldrich). The chromogen present was 3,3′-diaminobenzidine tetrahydrochloride, followed by counterstaining with hematoxylin [38].

### Statistical analysis

SPSS version 26 was used to analyze the data. Parametric tests were applied after confirming normality using the Shapiro-Wilk test, with results presented as mean ± standard deviation (SD). The parametric data were compared using a one-way analysis of variance (ANOVA) and Tukey’s test. A statistically significant *P* value of less than 0.05 was evaluated. Pearson correlation coefficients were calculated to assess linear relationships between different variables. A correlation coefficient (r) of -1 indicates a perfect negative correlation, while a coefficient of 1 indicates a perfect positive correlation. These correlations were used to evaluate associations between various parameters within the study.

### Computer-aided digital image analysis (digital morphometric investigation)

Slides were photographed with a MEIJI MX5200L microscope equipped with 20X and 40X lenses and an MVV5000CL digital eyepiece with a 5.0MP sensor. The resulting 20X images were analyzed using Fiji ImageJ (version 1.51r; NIH) software on an Intel Core i7-based computer. The Color Deconvolution 2 plugin was used to calculate the percentage of stained surface area. Each tissue slide had five random fields evaluated.

## RESULTS

### Pro and anti-inflammatory cytokines levels across different groups

The administration of AgNPs in Group III significantly increased the serum levels of IL-1β, TNF-α, IL-6, and IL-10 compared to Group I. Specifically, the levels of IL-1β, TNF-α, IL-6, and IL-10 in Group III were 208.17 ± 5.14 pg/ml, 89.45 ± 0.87 pg/ml, 84.99 ± 0.65 pg/ml, and 806.43 ± 6.17 pg/ml, respectively, whereas in Group I, these levels were 78.25 ± 4.95 pg/ml, 43.81 ± 0.71 pg/ml, 22.94 ± 0.78 pg/ml, and 324.27 ± 7.97 pg/ml, respectively (Table 1). Administration of *W. somnifera* significantly decreased serum IL-1β, TNF-α, IL-6, and IL-10 cytokines levels in Group V compared to Group III (Table 1).

**Table 1:** Serum pro and anti-inflammatory markers across different groups

	Control negative Group I	Group II	Group III	Group IV	Group V
**IL-1 (Pg/ml)**	78.25 ± 4.95	80.1 ± 2.09	208.17 ± 5.14^*#^	207.73 ± 2.89^*#^	82.8 ± 4.99^¶€^
**TNF-α (Pg/ml)**	43.81 ± 0.71	44.31 ± 0.46	89.45 ± 0.87^*#^	89.47 ± 0.63^*#^	46.52 ± 1.11^*#¶€^
**IL-6 (Pg/ml)**	22.94 ± 0.78	23.55 ± 0.58	84.99 ± 0.65^*#^	85.46 ± 0.45^*#^	34.61 ± 1.77^*#¶€^
**IL-10 (Pg/ml)**	324.27 ± 7.97	324.67 ± 6.02	806.43 ± 6.17^*#^	806.53 ± 8.36^*#^	408.00 ±3 .78^*#¶€^

Data expressed as mean ± SD; SD, standard deviation; P, Probability; Test used: One way ANOVA followed by post-hoc Tukey; *P <0.05 vs. Group I; *# P* <0.05 vs. Group II; *¶ P* <0.05 vs. Group III; *€ P* <0.05 vs. Group IV

### Serum liver biomarkers levels across study groups

**Figure 2:**
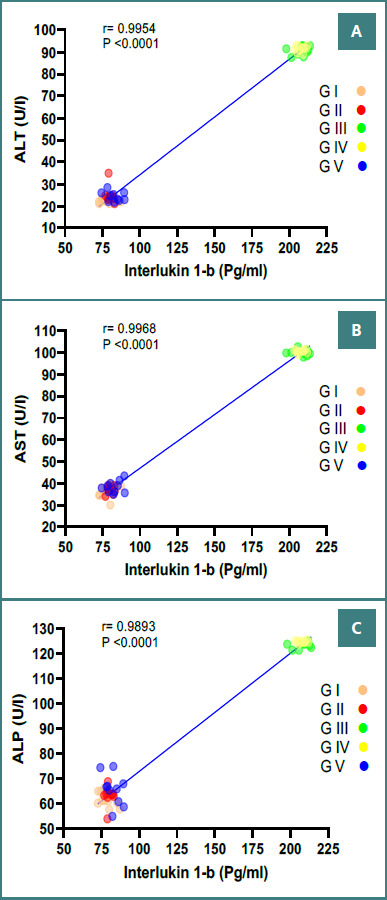
Correlation between serum IL-1β levels and liver enzyme activities in mice across different experimental conditions. The solid lines represent the linear regression and correlation coefficient (r). P is the significance level of correlation. The total number of subjects in each group was 10 mice. A, Correlation between Serum IL-1β and ALT (U/l); B, Correlation between Serum IL-1β and AST (U/l); C, Correlation between Serum IL-1β and ALP (U/l).

The administration of AgNP in Group III significantly increased serum liver enzyme levels: ALT (90.74 ± 2.10 U/l), AST (99.95 ± 1.33 U/l), and ALP (123.41 ± 1.30 U/l) compared to the control Group I (ALT: 22.51 ±1.38 U/l, AST: 36.25 ±3.01 U/l and ALP: 61.69 ± 2.94U/l, respectively). In addition, total protein (TP), albumin (ALB), total bilirubin (TBIL), and gamma-glutamyl transferase (GGT) were significantly different in Group III compared to Group I. However, Group V, treated with *W. somnifera*, showed a reduction in liver enzymes and significant improvements in TP, ALB, TBIL, and GGT compared to Group III (Table 2). Furthermore, a significant positive correlation was found between proinflammatory cytokine IL-1β and ALT, AST, and ALP (Figure 2).

**Table 2:** Serum biomarkers levels across different experimental models

	Group I	Group II	Group III	Group IV	Group V
**ALT (U/l)**	22.51 ± 1.38	24.71 ± 3.74	90.74 ± 2.10^*#^	91.50 ± 1.38^*#^	25.58 ± 3.52^¶€^
**AST (U/l)**	36.25 ± 3.01	37.05 ± 1.62	99.95 ± 1.33^*#^	100.54 ± 0.73^*#^	38.34 ± 2.74^¶€^
**ALP (U/l)**	61.69 ± 2.94	63.25 ± 3.69	123.41 ± 1.30^*#^	124.73 ± 0.47^*#^	65.75 ± 6.28^¶€^
**TP (g/dl)**	6.55 ± 0.12	6.52 ± 0.17	3.25 ± 0.12^*#^	3.22 ± 0.13^*#^	5.85 ± 0.19^*#¶€^
**ALB (µg/L)**	1.80 ± 0.05	1.78 ± 0.05	0.78 ± 0.08^*#^	0.79 ± 0.06^*#^	1.44 ± 0.10^*#¶€^
**TBIL (mg/dl)**	0.196 ± 0.015	0.234 ± 0.019	1.060 ± 0.078^*#^	1.068 ± 0.093^*#^	0.396 ± 0.043^*#¶€^
**GGT (IU/l)**	1.75 ± 0.07	1.74 ± 0.10	4.82 ± 0.08^*#^	4.82 ± 0.09^*#^	2.11 ± 0.09^*#¶€^

ALT, alanine aminotransferase; AST, aspartate aminotransferase; ALP, alkaline phosphatase; TP, Total protein; ALB, Albumin; TBIL, total bilirubin; GGT, Gamma-glutamyl Transferase; Data expressed as mean ± SD; Test used: One way ANOVA followed by post-hoc Tukey; *P <0.05 vs. Group I; *# P* <0.05 vs. Group II; *¶ P* <0.05 vs. Group III; *€ P* <0.05 vs. Group IV

### Antioxidant activities and oxidative stress markers in liver homogenate

Group III showed significantly decreased activities of antioxidant enzymes SOD, GSH-Px, and CAT and increased levels of oxidative stress markers MDA and PC compared to Group I. When AgNPs were injected, they were dose-dependent, with notable alterations at 35 mg/kg/bw. Specifically, SOD levels were 26.08 ± 0.41 U/mg in Group III compared to 46.22 ± 0.75 U/mg in Group I (*P* <0.05). GSH-Px activity was 43.85 ± 0.45 U/mg in Group III, significantly lower than 93.52 ± 0.50 U/mg in Group I. CAT activity in Group III was 40.84 ± 0.68 U/mg, significantly less than 86.37 ± 1.53 U/mg in Group I. MDA and PC levels were significantly higher in Group III (9.79 ± 0.15 U/mg and 0.371 ± 0.028 nmol/mg, respectively) compared to Group I (3.72 ± 0.12 U/mg and 0.0217 ± 0.001 nmol/mg, respectively) (Table 3). Treatment with *W. somnifera* (at a dose of 80 mg/kg/bw) in Group V significantly increased the activities of SOD, GSH-Px, and CAT and decreased the levels of MDA and PC compared to Group III (*P* <0.05, Table 3). No significant changes in antioxidant activities were observed in the saline-treated Group IV. Additionally, there were no signs of agitation, weight loss, sores, or mortality in Group II that received *W. somnifera* treatment.

**Table 3:** Activities of SOD, GSH-Px, CAT, MDA and PC levels in the liver homogenate of mice across different groups

	Group I	Group II	Group III	Group IV	Group V
**SOD (U/mg protein)**	**46.22 ± 0.75**	**46.23 ± 0.63**	26.08 ± 0.41^*#^	26.14 ± 0.50^*#^	42.02 ± 0.76^*#¶€^
**GSH-Px (U/mg protein)**	**93.52 ± 0.50**	**93.89 ± 0.82**	43.73 ± 0.61^*#^	43.85 ± 0.45^*#^	88.54 ± 0.94^*#¶€^
**CAT (U/mg protein)**	**86.37 ± 1.53**	**86.62 ± 1.12**	40.84 ± 0.68^*#^	40.81 ± 0.81^*#^	76.10 ± 1.18^*#¶€^
**MDA (U/mg protein)**	**3.72 ± 0.12**	**3.73 ± 0.12**	9.79 ± 0.15^*#^	9.81 ± 0.18^*#^	4.08 ± 0.11^*#¶€^
**PC (nmol/mg protein)**	**0.030 ± 0.004**	**0.031 ± 0.003**	0.371 ± 0.028^*#^	0.378 ± 0.039^*#^	0.075 ± 0.012^*#¶€^

Data expressed as mean ± SD; SD, standard deviation; P, Probability; Test used: One way ANOVA followed by post-hoc Tukey; *P <0.05 vs. Group I; *# P* <0.05 vs. Group II; *¶ P* <0.05 vs. Group III; *€ P* <0.05 vs. Group IV

### Histopathological results (light microscopic examination of H&E-stained sections)

**Figure 3:**
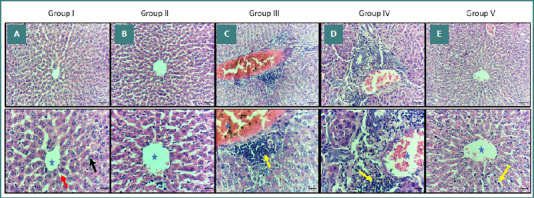
Histopathological examination (H&E staining) of liver sections from different experimental groups. A, B, liver section from Group I & II showing normal liver architecture, central vein (blue star), sinusoids (red arrow) and hepatocytes (black arrow). C, D, Liver section from Group III & IV showing congested central vein filled with RBCs, necrosis of hepatocytes, and inflammatory cells infiltration (yellow arrows). E, Liver section from Group V showing minimal central vain congestion, and insignificant inflammatory cells infiltration. Original magnification of upper raw ×200 and lower raw 400X.

Microscopic examination of H&E-stained liver sections revealed distinct histopathological changes among the experimental groups. Group I and Group II showed normal liver architecture, central veins, sinusoids, and hepatocytes (Figure 3AB). In contrast, Groups III and IV (AgNPs and AgNPs-treated saline groups) showed severe liver damage with congested sinusoids filled with red blood cells (RBCs) and hepatocyte necrosis, as well as dense inflammatory infiltrate and fibrous expansion of the portal tract (Figure 3 CD). Group V, which received both AgNPs and *W. somnifera* treatment, showed mild hydropic degeneration of hepatocytes and dilated congested sinusoids filled with RBCs (Figure 3E).

### Immunohistochemical results (light microscopic detection of NF-κB)

**Figure 4:**
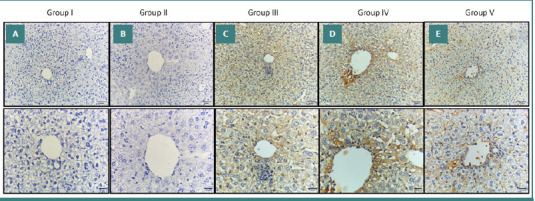
Immunohistochemical staining of NF-κB in liver sections from different experimental groups. A, B, Liver section from Groups I and II showing negative expression (no immune-reactive cells). C, D, Liver section from Groups III & IV showing diffuse strong expression of hepatocytes. E, Liver section from Group V showing weak expression in hepatocytes. Original magnification of upper raw ×200 and lower raw 400X.

Immunohistochemical staining for NF-κB revealed differential expression patterns in liver sections across the experimental groups. Group I and Group II showed normal liver specimens with negative NF-κB expression in hepatocytes (Figure 4AB). In contrast, Groups III and IV had positive NF-κB expression in hepatocytes, indicating activation of inflammatory pathways in response to AgNP exposure (Figure 4CD). In the AgNPs and *W. somnifera*–treated group (Group V), the liver showed positive, patchy (focal) NF-κB immunostaining in hepatic cells (Figure 4E). There was a significant increase in the NF-κB immunohistochemical positive area percentage in Group III compared to the control group, whereas Group V showed a significant decrease compared to Group III (Table 4).

**Table 4:** The quantified comparison of immunohistochemical staining in mice different groups

	Group I	Group II	Group III	Group IV	Group V
**NF-κB IHC positive area percentage**	1.32 ± 1.29	1.50 ± 1.32	54.94 ± 4.96^*#^	48.25 ± 4.46^*#^	28.68 ± 2.56^*#¶€^
**VEGF IHC positive area percentage**	0.89 ± 0.69	0.88 ± 0.77	56.41 ± 3.77^*#^	56.57 ± 4.35^*#^	17.06 ± 4.09^*#¶€^

Data expressed as mean ± SD; SD, standard deviation; P, Probability; Test used: One way ANOVA followed by post-hoc Tukey; *P <0.05 vs. Group I; *# P* <0.05 vs. Group II; *¶ P* <0.05 vs. Group III; *€ P* <0.05 vs. Group IV

### Immunohistochemical results (light microscopic detection of VEGF)

**Figure 5:**
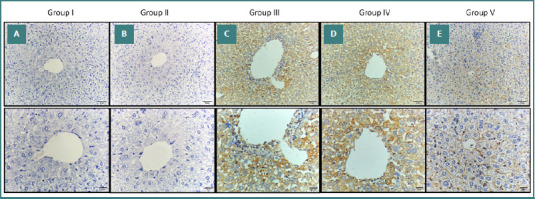
Immunohistochemical staining VEGF in liver sections from different experimental groups. A, B, Liver section from Groups I and II showing negative expression (no immune-reactive cells). C, D, Liver section from Groups III and IV showing diffuse strong expression of hepatocytes. E, Liver section from Group V showing weak expression in hepatocytes. Original magnification of upper raw ×200 and lower raw 400X.

Immunohistochemical analysis of VEGF expression in liver tissues revealed distinct patterns across different experimental groups. Liver sections from the control group and the *W. somnifera*–treated group (Group II) exhibited a normal appearance of liver specimens with negative expression of VEGF in the hepatocytes (Figure 5AB). In the AgNPs and AgNPs-treated saline groups (Groups III and IV, respectively), the liver showed positive expressions of VEGF in the hepatocytes (Figure 5CD). Additionally, in the AgNPs and *W. somnifera*–treated Group V, the liver showed a positive, patchy (focal) VEGF immunostaining in the hepatic cells (Figure 5E). There was a significant increase in the VEGF immunohistochemical positive area percentage of Group III compared to Group I. There was also a significant decrease in VEGF immunohistochemical positive area percentage in Group V compared to Group III (Table 4).

## DISCUSSION

The increasing use of metallic nanoparticles (MNPs) in various industries necessitates a thorough understanding of their potential impact on biological systems, especially concerning liver health. The liver, a crucial organ for detoxification, is particularly vulnerable to damage from these nanoparticles. AgNP-induced hepatic damage, which may lead to liver failure, is responsible for high rates of morbidity and mortality [39]. Studies have consistently shown a strong correlation between elevated serum levels of inflammatory mediators and the toxicity induced by MNPs in different animal models [40-43]. However, inhibiting inflammation could potentially decrease liver injury [44]. The quantification of enzymes in the blood is useful for determining the severity of hepatocellular damage [45].

In this study, the administration of AgNPs to mice (Group III) resulted in a significant increase in serum levels of IL-1β, TNF-α, IL-6, and IL-10 compared to the control group (Group I). These results align with Murphy *et al*. [46], who revealed that AgNPs can significantly upregulate the proinflammatory cytokine gene expression. Similarly, Ashour *et al*. [47] noted that nanoparticles, including AgNPs, induce inflammatory immune responses, with chemokines being major cytokines elevated at lower concentrations and during the early stages of exposure [47].

The administration of *W. somnifera* extract (Group V) for 14 days significantly reduced the serum levels of IL-1β, TNF-α, IL-6, and IL-10 compared to the AgNPs-intoxicated group (Group III) (*P* < 0.001) (Table 1). This finding is consistent with the research by Khan *et al*. [48], who demonstrated that *W. somnifera* aqueous extract effectively inhibits proinflammatory cytokine activity, including TNF-α, IL-1β, IL-6, and MMP-8, in collagen-induced arthritic rats. This can be attributed to the anti-inflammatory activities of the aqueous extract of *W. somnifera* roots. Similarly, Naidoo *et al*. [49] reported that *W. somnifera* could modulate cytokine production associated with cancer cachexia, suggesting that it may offer therapeutic benefits in reducing inflammation. The ability of *W. somnifera* to decrease proinflammatory cytokine levels and increase cancerous cell death may decrease the development and progression of cancer and cachexia. Moreover, our results support those of Minhas and Bhatnagar [50], who mentioned that *W. somnifera* root powder (500 mg and 1,000 mg per kg body weight) inhibits the proinflammatory cytokines in serum.

We observed significant increases in ALT, AST, and ALP liver enzymes and significant alterations in TP, ALB, TBIL, and GGT levels. These were induced by three successive days of AgNPs (35 mg/kg/bw) administration compared to the control group, which exhibited normal levels. The increased liver enzyme serum markers in the AgNPs-intoxicated mice in Group III indicate a deterioration of hepatic functions due to liver membrane damage resulting from silver toxicity, which led to the release of these enzymes into the blood. These results are supported by the data of Xu *et al*. [51], who demonstrated that the 'Ag + effect’ and 'particle-specific effect’ originate from AgNPs and contribute to the overall cytotoxicity of hepatic cells. Our study also agrees with Ramadan and Ghareeb [52], who stated that experimental rats injected with AgNPs showed higher levels of ALT, AST, and ALP liver enzymes than the control group. Moreover, our results support El-Naggar *et al*. [53], who showed that the total protein and albumin levels significantly (*P* <0.05) decreased in mice injected with AgNPs compared to their values in control mice.

In this study, we observed that administering *W. somnifera* for 14 consecutive days (80 mg/kg/bw) in Group V significantly mitigated liver damage induced by AgNPs, as evidenced by improved liver function markers (Table 2). This suggests that *W. somnifera* treatment offers protective effects against AgNPs-induced hepatic damage. The membrane-stabilizing activity of *W. somnifera* may be responsible for inhibiting intracellular enzyme leakage in AgNPs-intoxicated serum. These findings are supported by the study of Sultana *et al*. [54], which demonstrated that *W. somnifera* extract restored normal serum AST and ALT levels in gentamicin-intoxicated rats. This protective effect is likely attributed to its free radical scavenging activity, highlighting the potential of *W. somnifera* as a hepatoprotective agent.

In our study, significant decreases (*P* < 0.05) in SOD, GSH-Px, and CAT levels were observed in the liver homogenates of mice in Group III following AgNPs administration at 35 mg/kg/bw, compared to Group I (Table 3). These results are consistent with Olugbodi *et al*. [55], who similarly reported reduced levels of GSH, CAT, and SOD levels after subcutaneous AgNPs administration. Their study highlighted that AgNPs induced oxidative stress and compromised hepatic function.

Furthermore, our findings are consistent with those of Ansar *et al*. [56], who demonstrated that administration of AgNPs led to decreased SOD, CAT, and GSH levels and increased MDA levels in the livers of rats, indicating oxidative damage. Similarly, Zhao *et al*. [57] reported that AgNPs induced oxidative stress by reducing CAT activity and increasing MDA levels. The measurement of MDA content has long been used as a lipid peroxidation marker in studies related to oxidative stress and redox signaling. Our findings show that mice in Group III had higher levels of total lipid hydroperoxide as represented by MDA levels than Groups I and II (*P* <0.05) (Table 3). Moreover, consistent with several reports [58-61], our study also found increased PC levels in Group III compared to the control Group I. These results highlight oxidative stress as a crucial mechanism underlying the cytotoxic effects of AgNPs, which promote the generation of free radicals, ROS, and lipid peroxidation, ultimately leading to protein carbonylation.

The results of this study showed that *W. somnifera*, at a dose of 80 mg/kg/bw in Group V, determined a significant increase SOD, GSH-Px, and CAT levels compared to Group III (Table 3) (P<0.05). These findings are consistent with Sabina *et al*. [62], who suggested that *W. somnifera* exhibits hepatoprotective and antioxidant effects in rats intoxicated with acetaminophen, likely through its antioxidant properties, which enhance defenses against oxidative stress involving CAT, GSH-Px, and SOD enzymes. Similarly, Gupta *et al*. [63] reported that treatment with *W. somnifera* successfully attenuated GPx activity and inhibited lipid peroxidation in a dose-dependent manner. *W. somnifera* inhibited both the lipid peroxidation and protein oxidative modification induced by copper. Furthermore, our results align with Saggam *et al*. [64], who highlighted that withanolide-rich fractions from *W. somnifera* root methanolic extracts restored normal liver enzyme levels in rat models of drug-induced hepatic cytotoxicity. In addition, the saline-treated mice in Group IV did not show any improvement concerning liver enzymes.

In the present study, the AgNP-intoxicated Group III had congested liver sinusoids filled with RBCs, hepatocyte necrosis, dense inflammatory infiltrate, and fibrous expansion of the portal tract (Figure 3CD). These findings are consistent with Hamad *et al*. [65], who noted vascular congestion in AgNP-treated mice, characterized by dilated blood vessels filled with RBCs, hydropic changes, and vacuolar degeneration. Additionally, our findings support those of Yaqup *et al*. [66], who reported that at higher AgNPs dosing rates, the liver hepatocytes of AgNPs-exposed mice gradually distorted, with some cells exhibiting increasing degrees of necrosis and apoptosis, leading to cell detachment and enlarged nuclei. The central vein was damaged and slightly widened due to the liver’s increased oxidative stress. The size of the hepatocytes also appeared to be growing. Al-Doaiss *et al*. [67] also observed granular eosinophilic hepatocytes with ground glassy cytoplasm and occasional necrotic and apoptotic hepatocytes in mice administered 10 nm AgNPs, along with sinusoidal dilatation and inflammatory cell infiltration.

However, in Group V, the liver showed mild hydropic degeneration of hepatocytes and dilated congested sinusoids filled with RBCs (Figure 3E). This aligns with Vasavan *et al*. [68], who noted the potential of *W. somnifera* roots to reverse liver tissue damage. These findings are indicative of the hepatic protection offered by *W. somnifera* root. The *W. somnifera* group showed mild congestion, with few pyknotic nuclei and necrosis. The results support Sabina *et al*. [62], who reported similar mild degeneration and less disarrangement of hepatocytes in *W. somnifera*-treated rats. Moreover, our results are consistent with those of Malik *et al*. [69], who found that treatment with *W. somnifera* root extract preserved liver architecture with only mild hepatocyte degeneration, comparable to control groups. These observations highlight the hepatoprotective potential of *W. somnifera*, likely due to its antioxidant properties. Additionally, Anwar *et al*. [23] reported that treatment with *W. somnifera* in combination with AgNPs resulted in mild vacuolation in the centrilobular area, along with a dilated central vein and portal tract, further supporting the protective role of *W. somnifera* against liver damage.

The pleiotropic transcription factor NF-κB works as one of the key supervisors for triggering and amplifying inflammation [70,71]. Luedde and Schwabe [72] suggested that it may be possible to target NF- κB to prevent or treat liver damage. In Groups III and IV, the liver showed positive diffusing strong expressions of NF-κB in hepatocytes (Figure 4CD) compared to Groups I and II, which exhibited negative expressions of NF-κB in the hepatocytes (Figure 4AB). In Group V, where mice were treated with *W. somnifera* (80 mg/kg/bw) following AgNP exposure, NF-κB immunostaining was patchy and weak (Figure 4E). This indicates a significant reduction in NF-κB activity compared to the AgNPs-only group (Group III). Our findings align with the work of Martorana *et al*. [73], who demonstrated that withaferin A, a key compound in *W. somnifera*, can effectively reduce NF-κB activity and subsequent production of inflammatory markers such as TNF-α, COX-2, and iNOS, which are induced via the LPS/TLR4 pathway. Additionally, withaferin may be a viable candidate for treating neuroinflammatory and stress-related conditions, according to Martorana *et al*. Moreover, Borgmann *et al*. [74] mentioned that a water extract of *W. somnifera* reduced the gene expression of both CCL2 and CCL5 through TNF-α, which was mediated by inhibiting NF-κB activity. Furthermore, the findings of Sikandan *et al*. [75] are consistent with our findings and demonstrate that the anti-inflammatory effect of *W. somnifera* may be due to its ability to suppress the NF-κB and mitogen-activated protein kinase pathways and modulate cytokine expression. These results suggest that *W. somnifera* can potentially protect against inflammation.

VEGF plays a crucial role in angiogenesis, essential for tissue healing and organ repair [76]. In our study, the expression of VEGF in the liver was significantly increased in the AgNPs and AgNPs-treated saline groups (Groups III and IV, respectively), with positive immunostaining observed in hepatocytes (Figure 5CD). In contrast, the group treated with *W. somnifera* (Group V) showed weaker immunostaining for VEGF (Figure 5E). These findings align with research by Saha *et al*. [77], which suggests that withaferin A, a compound found in *W. somnifera*, may interact effectively with VEGF as an anti-VEGF agent. Similarly, Xu *et al*. [78] demonstrated that withaferin A reduces VEGF levels, supporting our observation of decreased VEGF expression in the *W. somnifera*-treated group. Furthermore, Sari *et al*. [79] investigated the effects of WA/CAPE (WACAPE) on VEGF signaling and found that WACAPE treatment significantly downregulated VEGF mRNA levels, highlighting the potential of withaferin A in modulating VEGF expression. The study used a limited sample size, with just 50 male Swiss albino mice divided into five groups.

Like many studies, this investigation has several limitations that may impact the robustness and generalizability of the findings. Firstly, the relatively small sample size used in this study limits the ability to generalize the results across a broader population. Secondly, the 14-day duration of the study may not have been sufficient to fully capture the long-term effects of *W. somnifera* treatment and AgNP toxicity. Extended research periods may yield a more thorough comprehension of the results. Furthermore, this study did not explore the dose-response relationships of *W. somnifera* in mitigating AgNP toxicity. Understanding the optimal dosage and its effects could offer valuable insights into the medicinal potential of *W. somnifera*. Overall, the study offers insightful information about the impact of this valued medicinal plant.

## CONCLUSION

In traditional medicine, *Withania somnifera*, commonly known as ashwagandha, is recognized for its diverse pharmacological properties, including anti-convulsive, anti-tumor, anti-inflammatory, immunostimulant, antidepressant, and antioxidant activities. In the current study, we observed significant reductions in AgNP-induced liver toxicity by administering *W. somnifera* root extract, as evidenced by improved liver biomarkers, cytokine levels, and histopathological changes. This study recommends the development of *W. somnifera* as a novel therapeutic agent or functionally coadjuvant for the prevention of toxicity-mediated inflammation or liver diseases. Given its historical use and the promising results obtained, further research is warranted to explore the optimal dosage, long-term effects, and mechanisms of action of *W. somnifera*. This would enhance our understanding of its therapeutic potential and support its development as a viable treatment for liver damage and other related conditions.

## Data Availability

The data supporting the findings of this study are available from the corresponding author, M.F. El-Refaei, upon reasonable request. The data are not currently accessible to the general public but will be provided on demand.

## Personal thanks

We appreciate Dr. Mai Abd-Elwahab of the Zagazig University Faculty of Medicine’s help with the histopathological portion of this study.
